# Identification of donor microbe species that colonize and persist long term in the recipient after fecal transplant for recurrent *Clostridium difficile*

**DOI:** 10.1038/s41522-017-0020-7

**Published:** 2017-06-07

**Authors:** Ranjit Kumar, Nengjun Yi, Degui Zhi, Peter Eipers, Kelly T. Goldsmith, Paula Dixon, David K. Crossman, Michael R. Crowley, Elliot J. Lefkowitz, J. Martin Rodriguez, Casey D. Morrow

**Affiliations:** 1 Biomedical Informatics, Center for Clinical and Translational Sciences, University of Alabama at Birmingham, Birmingham, Alabama 35294 USA; 2 Department of Biostatistics, University of Alabama at Birmingham, Birmingham, Alabama 35294 USA; 3 Department of Cell, Developmental and Integrative Biology, University of Alabama at Birmingham, Birmingham, Alabama 35294 USA; 4 Department of Genetics and Heflin Center for Genomic Science, University of Alabama at Birmingham, Birmingham, Alabama 35294 USA; 5 Department of Medicine, Division of Infectious Diseases, University of Alabama at Birmingham, Birmingham, Alabama 35294 USA; 6 Department of Microbiology, University of Alabama at Birmingham, Birmingham, Alabama 35294 USA

## Abstract

Fecal microbiota transplantation has been shown to be an effective treatment for patients with recurrent *C. difficile* colitis. Although fecal microbiota transplantation helps to re-establish a normal gut function in patients, the extent of the repopulation of the recipient microbial community varies. To further understand this variation, it is important to determine the fate of donor microbes in the patients following fecal microbiota transplantation. We have developed a new method that utilizes the unique single nucleotide variants of gut microbes to accurately identify microbes in paired fecal samples from the same individual taken at different times. Using this method, we identified transplant donor microbes in seven recipients 3–6 months after fecal microbiota transplantation; in two of these fecal microbiota transplantation, we were able to identify donor microbes that persist in recipients up to 2 years post-fecal microbiota transplantation. Our study provides new insights into the dynamics of the reconstitution of the gastrointestinal microbe community structure following fecal microbiota transplantation.

*Clostridium difficile* (*C. difficile*) is the major causative agent for infective antibiotic associated diarrhea.^1, 2^ Infections acquired in healthcare settings have estimated health care costs in the billions.^3, 4^ Standard treatments for *C. difficile* infection consist of metronidazole, vancomycin, or fidaxomicin, which result in a 20% rate of recurrence; after a third recurrence, the risk of further episodes is even higher.^5^

When antibiotic therapy fails for the patients, fecal microbiota transplantation (FMT) has had remarkable success rates of greater than 90% for alleviation of symptoms and restoration of health.^6^ Numerous studies have characterized the microbial composition of the recipient following transplant.^7–11^ Although the microbe composition of the recipient post transplant was different after FMT, the similarity of the reconstituted microbe community to that of the donor varied between different patients.^7–11^ The significance of these differences as they relate to the long-term stability of the FMT re-constructed gut microbe community is unknown. As a first step to understanding the microbial ecology of the reconstituted community, it is necessary to determine the fate of the donor microbes after FMT. A recent study by Li et al. has shown coexistence of donor microbes in recipient post FMT in metabolic syndrome patients.^12^ Their method uses a comparison of single nucleotide variants (SNV) of donor, pre-FMT and post FMT samples to show the presence of donor microbes in post FMT samples. However, the pre-FMT samples of patients with *C. difficile* infection have been exposed to several rounds of antibiotics resulting in a near depletion of commensal gut microbes.^7–11^ Thus, it was not practical to use the method by Li et al. to determine the presence of donor microbes in patients post-FMT. Recently, Schloissnig et al., used metagenomic sequence analysis of microbe species in the human microbiome to demonstrate that individuals have their own distinct SNV that were stable for up to 1 year.^13^ In this study, we have exploited the unique SNVs of gut microbes of individuals and developed a method to establish whether or not paired donor and recipient post-FMT samples share the same unique SNVs across the genome. Our findings demonstrate the colonization and persistence of certain donor microbial species in the recipient post-FMT in *C. difficile* patients.

We first constructed a reference sequence of 93 microbial species, commonly found in healthy and FMT samples (Supplementary Table 1): 71 most abundant species from the healthy microbiome were selected from Schloissnig et al. (accounting for 99% of the aligned HMP data) and the remaining 22 were microbes found to be abundant in recipients.^13^ To calculate the SNV, the metagenome is first mapped on to the reference sequences. Raw data obtained either from the NIH Human Microbiome Project or our study was aligned to the reference sequence using Burrows–Wheeler Aligner, and multi-sample SNV calling was performed using Genome Analysis Toolkit (version 3.6).^14, 15^ Then, for a given species, a pairwise comparison was performed between two samples to measure their genome-wide SNV similarity. Regions of low read coverage, sequence repeats, indels, and structural variants cause alignment difficulties that may also produce increased numbers of (false) SNVs. Therefore, to minimize the effect of these clustered SNVs on overall genome wide similarity, we developed a window-based SNV comparison approach, window-based SNV similarity (WSS), which measures SNV variation between two samples (Fig. 1a). The resolution of WSS is not at the level of single nucleotide variants, but is based on the chosen window size. This window-based comparison is less sensitive to genomic regions that may have artificially large numbers of SNVs, and therefore provides a more accurate determination of the true variability that exists between species (i.e., representing potentially strain differences) of two samples (see Supplementary Data for further explanation).

**Fig. 1:**
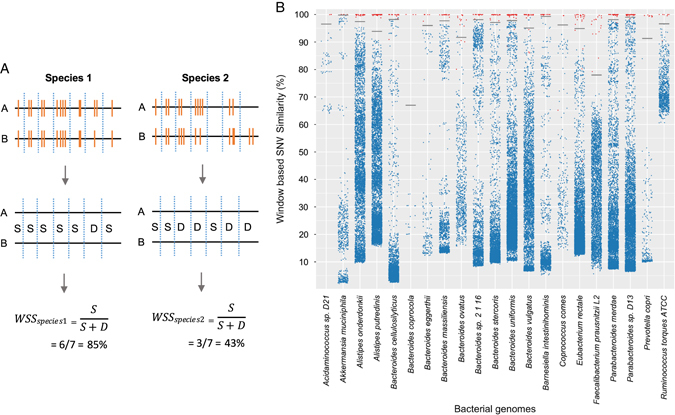
Window-based SNV similarity for pair wise sample comparisons. **a** Schemtatic of window-based SNV similarity (WSS). WSS method used to determine the similarity of two samples (**a** and **b**) for two different species (Species 1 and 2). The metagenome DNA sequences were aligned to reference genomes to call SNVs. INDELS were not included in the analysis. To calculate the WSS score, the reference genome is divided into sequential, non-overlapping 1 KB windows. A window is defined as similar if the SNV pattern (denoted as *orange bars*) is exactly the same between two samples for that region or if no SNV (S in the equation) is present in both samples; otherwise, the window is called dissimilar (denoted as D). The WSS score (as a percentage) is calculated for each pairwise comparison for every microbial species. Both sample pairs are required to have minimum coverage ≥ 20% and average depth ≥ 5 to be included for comparison. Low coverage windows with more than 50% of the bases having a read depth < 5 were ignored. The proportion of similar windows across the genome defines the genome-wide SNV similarity between two samples for a given species, and is referred to as the WSS score (see Supplementary Data for further explanation). **b** Scatter plot of the WSS score for all possible pairwise sample comparisons of selected genomes from the HMP data set. WSS scores for all pairwise sample comparisons of species detected in the HMP dataset were determined. The WSS scores (percent similarity) from unrelated HMP sample pairs are presented as *blue points*. The sample pairs from the same individual at different times (temporally linked) are displayed as *red dots*. The number of dots for a given species was proportional to the number of samples where the species is present (at depth > 5X). Although 93 species were analyzed, the results from the 21 species that were present in the FMT samples are shown. For each species, we modeled the WSS of related and non-related samples and constructed a simple binary classifier using logistic regression (Supplementary Data). From the classifier, we identified the WSS cutoff value that differentiates a related sample from a non-related sample (Supplementary Table 7)

We applied the WSS method to metagenomic data from a set of 136 metagenomes (51 individuals sampled once, 41 individuals sampled twice, and one individual sampled three times) from the NIH Human Microbiome Project (Supplementary Table 2). The raw data consisted of 1.3 terabases (13.6 billion raw sequence reads) of which 5.5 billion reads (41.5% of raw reads) aligned to the reference sequence allowing SNVs to be called. The WSS was calculated for all possible pairwise comparisons of samples for each microbial species, of which 21 selected species are shown in Fig. 1b and Supplementary Table 3. We found that samples from the same individual taken at two separate times (temporally related samples) have a distinctively higher WSS (i.e., SNV similarity) than non-related samples at the species level. Statistically, the related and unrelated pairs show a bimodal distribution with very little overlap (*p*-values range 0.007–4.7E-27; Mann–Whitney *U* Test). We applied a non-parametric test because even after applying a log transformation, the data still did not pass the Shapiro-Wilk test for normal distribution (Supplementary Table 4). Thus, the WSS for a sample pair can be used to predict whether two different samples are related based on their SNV similarity. For each species, we modeled the WSS of related and non-related samples and constructed a simple binary classifier using logistic regression (Supplementary Data). From the classifier, we identified the WSS cutoff value that differentiates a related sample from a non-related sample (Supplementary Table 7).

We applied the window-based SNV method to samples obtained from six donors and seven FMT recipients treated for *C. difficile* infection with the FMT samples obtained early post-FMT (1–6 months following transplant) when colonization of the gut with donor microbes should have occurred (one donor was used for two transplants, FMT F and G). Samples at 2 years post-FMT were available for two of the seven transplants and the recipient pre-FMT was available for four transplants. Five of the seven transplants were with un-related donors while two of the transplants were done with donors from spouses (Supplementary Table 5). The DNA from fecal samples was prepared, processed and sequenced, using the Illumina HiSeq2500 platform, with 100 base paired-end reads with an average of 38 million reads per sample (Supplementary Table 5). The species abundance was estimated using the computational tool MetaPhlan2 that relies on mapping whole genome sequence read data to a clade specific marker database.^16^ The microbe composition of the recipient’s pre transplant was dominated with microbes such as *Escherichia coli*, *Lactobacillus salivarius*, and *Klebsiella oxytoca* that were not found or were found in very low relative abundance, in the donors or in the recipients post transplant and were not used for subsequent pairwise comparisons (Supplementary Table 8). Using the MetaPhelan2 data at the species level, we generated a PCoA plot (Bray-Curtis) that show the relationship of the microbe communities of the donor and pre and post-FMT samples (Supplementary Data).

The WSS was calculated for all possible pairwise comparisons of FMT samples (all donor–donor, donor–recipient FMT and recipient FMT-recipient FMT combinations where there was sufficient genome coverage; Supplementary Table 6). The sample pairs were grouped in two categories. First is the FMT-related where there is a possibility of shared species (e.g., the donor–recipient pairs, DA and T1A in FMTA). The second category is FMT-unrelated where two samples should not share any common microbial species (e.g., the unrelated pairs, DB (in FMTB) and DA (in FMTA)). Using the classifier based on full HMP as a training dataset, we classified the FMT-related sample pairs into related (represent same SNV pattern) and unrelated (the sample pair does not share same SNV pattern), implying that sample pair might have different microbial species (Supplementary Table 7, Fig. 2a). The classifier correctly predicted that all FMT-unrelated samples (second category) were unrelated.

**Fig. 2:**
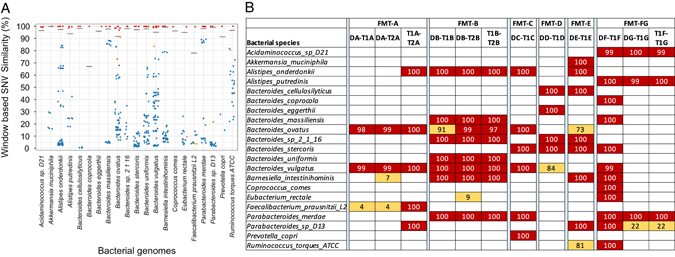
WSS Analysis for FMT donor–recipients. **a** Scatter plot of the pairwise sample comparisons of species from the FMT. WSS scores were calculated for all possible sample pairs (donor–donor, donor–recipient post-FMT, and recipient post FMT-recipient post FMT). The number of dots for species varies because not all samples contain every species (at depth cutoff > 5X). The *blue dots* represent FMT-unrelated sample pairs, whereas *red* and *orange points* represent FMT-related sample pairs (where both samples were derived from a particular FMT transplant). *Orange dots* represent related sample pairs with WSS scores below the boundary cutoff, and *red dots* represent related sample pairs with WSS scores above the boundary cutoff (see Supplementary Data). *Grey horizontal bars* mark the WSS score boundary cutoff derived from the HMP dataset (see Supplementary Table 7). **b** The WSS for different FMT donor–recipients post transplant. Donor and recipient post transplant samples from seven different FMT were analyzed (FMT A-F). DA-T1A refers to donor A compared to recipient FMT A at time 1; DA-T2A refers to donor A compared to recipient FMT at time 2. (These 2-year samples were only available for FMT-A and FMT-B). The transplant FMT-FG refers to two FMTs that used the same donor (DF, DG) for two separate recipients (F and G) FMT. The T1A-T2A and T1B-T2B are recipient-recipient comparisons at the two different time points. *Red shaded boxes* display WSS scores above the WSS boundary cutoff as determined by the classifier, suggesting shared microbial species between the two samples. The *orange shaded boxes* depict sample pairs where the WSS score was below the boundary cutoff. Empty boxes denote missing data that means sample pairs with sequence coverage or read depth too low to compare

For all pairwise comparisons of FMT-related samples that were predicted as related, the WSS were above the WSS classification boundary cutoff (all 97–100%; Fig. 2b), implying that the microbial species of the donor and recipient post transplant were related. *Bacteroides spp*. were identified as the most common donor microbe found in the recipients post transplant (in all except FMT-G) which may be explained by the finding that *Bacteroides* have evolved receptors for intestinal cells that facilitate retention in the gastrointestinal tract.^17^ *B. ovatus, B. stercoris, B. massiliensis*, *B. celluloslyticus*, *and B. vulgatus* were identified as having been transferred from the donor in multiple transplants while *B. eggerthii and B. uniformis* were each only found in one different transplant. Analysis of individual FMT highlighted the complexity of the microbial ecology of the gastrointestinal tract environment. For example, even though *B. stercoris* was transferred from the donor to recipient in FMT-E, the *B. ovatus* detected in the same donor and recipient post-FMT comparison was not similar. In the FMT-D transplant, the *B. vulgatus* of the donor and recipient was not similar; however, in this transplant *B. ergerthii, B. sp2116*, *B. stercoris*, and *B. celluloslyticus* were transferred from donor to recipient. Interestingly, in the study where the same donor was transplanted into separate recipients (FMT-FG), four *Bacteroides spp*. *(B. copracola, B. massillienis, B. stercoris and B. vulgatus*) in FMT-F were identified as similar to the donor (DF-T1F) while no *Bacteroides spp*. had sufficient sequence coverage for WSS score calculation in the FMT-G recipient (DG-T1G). This result could not be explained by a mechanical failure of the FMT-G transplant since *P. merdae, Acidaminococcus spD21*, and *A. putredinis* from the donor were identified in both FMT-F and in FMT-G.

An effective long-term stable transfer would require the microbes in the FMT to access, occupy, and possibly out-compete the resident recipient microbes for niche space in the gastrointestinal tract following transplantation.^18^ Therefore, we next examined the long-term persistence of the transplanted microbial communities after FMT through analysis of samples 2 years after transplant. We found identity between the donor and recipient post transplant (both early and 2 year samples) for *B. vugatus and B. ovatus* in the FMT-A transplant, and several *Bacteroiodes spp, A. onderkondii, P. merdae*, and *B. intestinihominis* for the FMT-B transplant. The demonstration that certain transplanted microbes can persist for up to 2 years demonstrates the potential of using FMT for long-term changes in the composition of the gastrointestinal tract microbe communities.

Finally, analysis of early and late samples from FMT-A revealed that the *F. prausnitzii* that was present at early and 2 years in FMT-A shared identity with each other but did not share identity with the transplant donor, even though the abundance of *F. prausnitzii* in the donor was high (Fig. 2b and Supplementary Table 8). It is possible these *F. prausnitzii* strains came from dormant microbes that had been sequestered in niches of the recipient (and thus not in the fecal samples), possibly as a result of the extensive antibiotics used in the *C. difficile* recipients.^19^ Alternatively, it is also possible that the new strain in the recipient was a minority strain in the donor sample and thus not detected in the donor, but was at a competitive advantage following in the recipient post FMT. Thus, as suggested by Li et al. where FMT was used in patients with metabolic syndrome, as yet undefined recipient differences, such as niche competition or availability, could impact the microbe composition of the transplant recipient.^12^

In summary, using a method that exploits the unique SNV patterns of gut microbes to distinguish individual strain SNV patterns, we demonstrate the presence and persistence of certain donor microbes in FMT recipients. The clinical success of FMT in treating *C. difficile* has prompted the consideration of FMT to be used for treatment of other gastrointestinal diseases.^12, 20^ The results of our analysis provides new insights into the ecology of the human gastrointestinal tract niches that are essential for development of new approaches to improve health via manipulation of this complex microbial community.^21^

## Data availability

All relevant data are available from the authors upon request. The HMP metagenomics data is obtained from HMP repository hosted at Amazon (https://aws.amazon.com/datasets/human-microbiome-project/). All gut samples were downloaded from amazon S3 (s3://human-microbiome-project/HHS/HMASM/WGS/stool/). The sequence data for the FMT are deposited in the sequence read archive under accession number SRP082182. The code repository used for analysis and processing of metagenomics is available at https://github.com/ranjit58/mgSNP.

## Electronic supplementary material

Supplementary Material

Supplementary Tables
