# Author Correction: Identification of donor microbe species that colonize and persist long term in the recipient after fecal transplant for recurrent *Clostridium difficile*

**DOI:** 10.1038/s41522-017-0037-y

**Published:** 2017-11-03

**Authors:** Ranjit Kumar, Nengjun Yi, Degui Zhi, Peter Eipers, Kelly T. Goldsmith, Paula Dixon, David K. Crossman, Michael R. Crowley, Elliot J. Lefkowitz, J. Martin Rodriguez, Casey D. Morrow

**Affiliations:** 1 Biomedical Informatics, Center for Clinical and Translational Sciences, University of Alabama at Birmingham, Birmingham, AL 35294 USA; 2 Department of Biostatistics, University of Alabama at Birmingham, Birmingham, AL 35294 USA; 3 Department of Cell, Developmental and Integrative Biology, University of Alabama at Birmingham, Birmingham, AL 35294 USA; 4 Department of Genetics and Heflin Center for Genomic Science, University of Alabama at Birmingham, Birmingham, AL 35294 USA; 5 Department of Medicine, Division of Infectious Diseases, University of Alabama at Birmingham, Birmingham, AL 35294 USA; 6 Department of Microbiology, University of Alabama at Birmingham, Birmingham, AL 35294 USA

**Correction to:** *npj Biofilms and Microbiomes* (2017); doi:10.1038/s41522-017-0020-7

The affiliation details were incorrect in this article. The correct affiliation details are given below:

^1^Biomedical Informatics, Center for Clinical and Translational Sciences, University of Alabama at Birmingham, Birmingham, AL 35294, USA; ^2^Department of Biostatistics, University of Alabama at Birmingham, Birmingham, AL 35294, USA; ^3^Department of Cell, Developmental and Integrative Biology, University of Alabama at Birmingham, Birmingham, AL 35294, USA; ^4^Department of Genetics and Heflin Center for Genomic Science, University of Alabama at Birmingham, Birmingham, AL 35294, USA; ^5^Department of Medicine, Division of Infectious Diseases, University of Alabama at Birmingham, Birmingham, AL 35294, USA and ^6^Department of Microbiology, University of Alabama at Birmingham, Birmingham, AL 35294, USA.

In addition, this article was originally published without the accompanying supplementary tables. This file is now available in the HTML version of the article; the PDF was correct from the time of publication.

