# Supplementary material for: Identification of donor microbe species that colonize and persist long term in the recipient after fecal transplant for recurrent Clostridium difficile
Source: NPJ Biofilms Microbiomes. 2017 Jun 7;3:12. doi: 10.1038/s41522-017-0020-7 (PMC5462795; doi:10.1038/s41522-017-0020-7)
Supplement: Supplementary file 1 — Supplementary Material [file 41522_2017_20_MOESM1_ESM.pdf]

Supplementary Data

Identification of Donor Microbe Species that Persist Long Term in the Recipient After Fecal Transplant for Recurrent *Clostridium difficile*

Ranjit Kumar, Nengjun Yi, Degui Zhi , Peter Eipers, Kelly T. Goldsmith, Paula Dixon, David K. Crossman, Michael R. Crowley, Elliot J. Lefkowitz , J. Martin Rodriguez and Casey D. Morrow

FMT donors and patients.

The times of collection for the seven FMT used for this study can be found in Supplementary Table 5. Four of the seven FMT were done with unrelated donors (note the donor in FMT- FG was used for two FMT recipients).

All the Donors were in good health and screened within 7 days of the planned FMT procedure for infections that may be transmitted by the FMT using the following tests:

a. Hepatitis A IgM antibody
b. Hepatitis B core antibody
c. Hepatitis B surface antigen
d. Hepatitis C antibody
e. HIV 1&2 antibody
f. *H. pylori* IgG antibody
g. Stool for *C. difficile* (LAMP)
h. Stool for ova & parasites

Recipients were tested for:

a. Hepatitis B surface antibody quantitative
b. Hepatitis B surface antigen
c. Hepatitis C antibody
d. HIV 1&2 antibody
e. Stool for *C. difficile* (LAMP).

Once screening was complete the Donor collected a stool sample in a standard closed container. The sample was processed and prepared in a laboratory in the Division of Infectious Diseases as previously described [1].

All patients in this study received FMT by nasogastric tubes (NG) performed by physicians with experience in placing NG. An X ray was obtained prior to the transplant to make

sure the tube was well positioned.

After the procedure the patients were followed up in clinic. All patients reported normal bowel movements at the time of follow up, and no clinical complications were observed at the times of fecal collection (1-6 months or 2 years).

Fecal sample collection.

A consent form was obtained for FMT and microbiome analysis as part of an ongoing institutional review board (IRB) approved study at the University of Alabama at Birmingham. Individual fecal samples were collected from donors (at the time of transplant) and recipients post FMT at the designated times. Samples were processed and archived as previously described [2].

DNA preparation.

The DNA sample from cryopreserved fecal samples (100 mg/ml) was prepared using the Zymo fecal DNA isolation kit [2].

DNA sequencing.

The DNA was sheared using a Covaris S series sonicator followed by standard DNA library prep using the NEBNext Ultra II kit (NEB) following the manufacturer's instructions. We obtained sufficient DNA from all donors and recipients post transplant (RpT). We obtained sufficient DNA from 4 of the 7 FMT (identified in Supplementary Table 8). The resulting library was size selected on a 1.8% agarose gel to isolate 400-600 base pair fragments. The DNA was sequenced on the HiSeq 2500 sequencer (Illumina) using paired-end 100bp SBS chemistry with established protocols. The numbers of sequence reads are found in Supplementary Table 5.

Processing of DNA sequences.

The sequencing protocol provides paired-end reads of length 100 bases. Quality control steps include trimming of adapters, filtering low quality reads (sliding window of 50 bases having QScore < 20), and filtering of short sequences (sequence length < 50 bases) using PRINSEQ

(http://prinseq.sourceforge.net/. Contamination filtering (human genome: hg19 reference sequence) is performed using bowtie2.

Taxonomic composition was assessed using MetaPhlan2 that relies on mapping WGS read data to a clade specific marker database [3] (Supplementary Table 8). We found that recipient before transplant had very low abundance of microbes in common with the donor or recipient post FMT (e.g. *Bacteroides*) that precluded these samples being analyzed in the pair wise comparisons.

Comparing WSS method with general SNP based analysis.

We ran a simulation on five most abundant species and performed a general SNP based analysis (where the WSS method is not included) and identified the similarity distribution between all pairs of metagenomes (each species separately). The distance metric (dissimilarity metric) was the count of SNP differences between two pair of samples. To compare with the WSS method later, we converted the dissimilarity matrix into similarity matrix and re-scaled it from 0-100. The distribution we obtained is shown in Supplementary Figure 1.

As expected, for each species, we found bimodal distribution for similarity (for related and unrelated sample pair). **Even though a small overlap between the distributions was observed, the classifier that was built had a significant misclassification rate.**

We also ran WSS calculation on the same dataset (shown in Supplementary Figure 2). In this case, we observed **smaller** overlap in the bimodal distribution compared to SNP method.

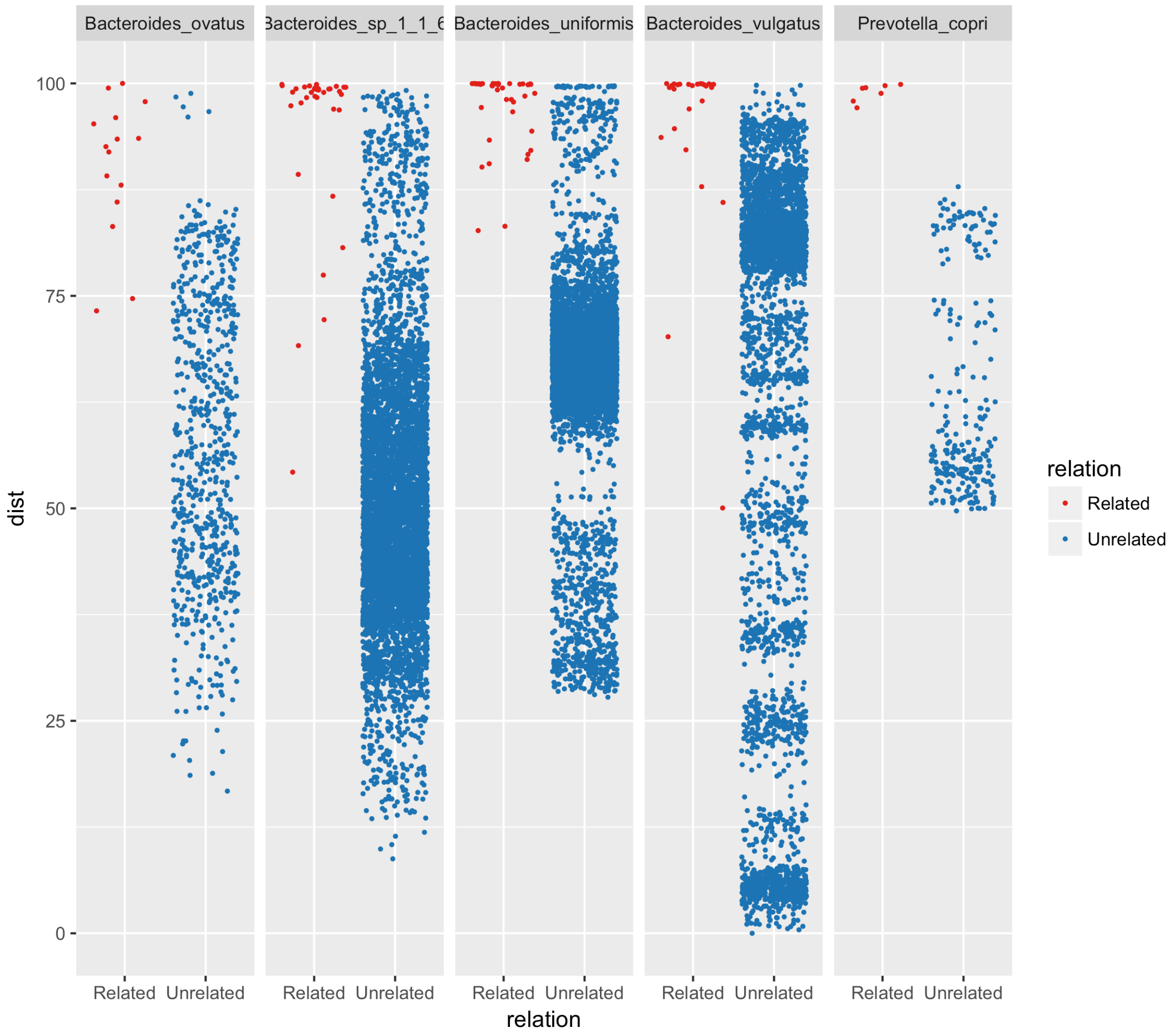

Supplementary Data Figure 1: Distribution of SNP based similarity score for related and unrelated sample pair for 5 species

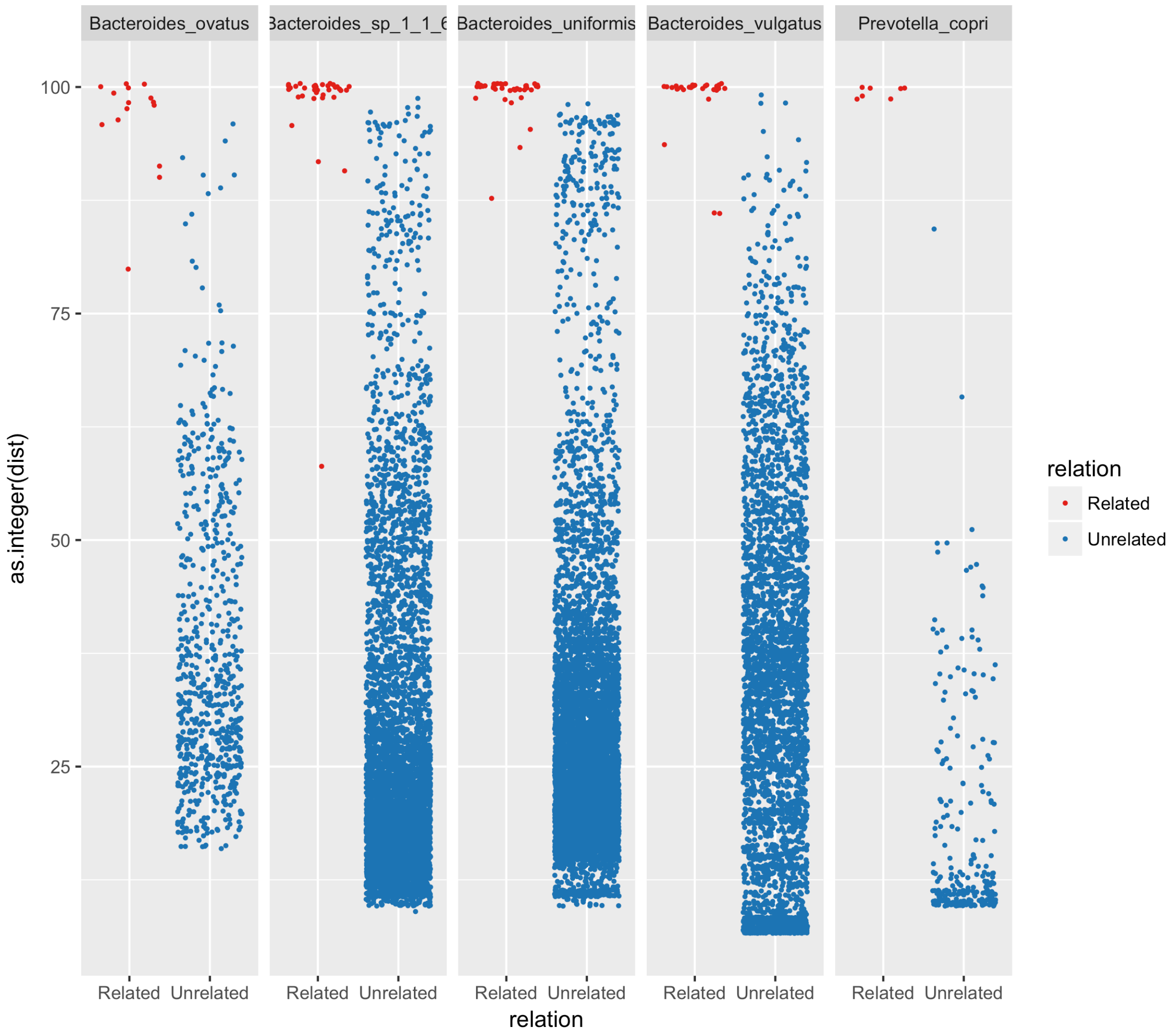

Supplementary Data Figure 2: Distribution of WSS (Window based similarity score) for related and unrelated sample pair for 5 species

We also compared using simulation the minimum error (False positive + False negative) what the classifier produces in both SNP method and WSS method. Based on simulation, the WSS method was more accurate as it shows small prediction error rate (below).

Similary Metric	Bacteroides_ovatus	Bacteroides_sp_1_1_6	Bacteroides_uniformis	Bacteroides_vulgatus	Prevotella_copri
SNP	9	20	39	13	0
WSS	3	4	3	3	0

Supplementary Data Table 1. Simulation of minimum number of error (False positive + False negative) the best case classifier would make using general SNP based method and WSS method. Lowest error rate for given species is colored in green.

Comparing different window size in WSS method.

We simulated different window size parameter (0.1 kb, 0.5kb, 1kb, 2kb, 5kb, 10kb, 100kb) in the WSS method for SNP profile similarity. To measure performance, we simulated and identified the minimum number of error (False positive + False negative) the best case classifier would make, provided in the Table 2. From the results it seems that larger window size (10 kb or more) are not optimal choice as they resulted in large number of errors. The smaller window size (0.1kb to 2kb) provided best and similar performance measure in given dataset, which means any window size in this range can be optimal choice. For our analysis, we have used a window size of 1kb as similar window sizes are used in previous studies [4].

Window_size(kb)	Bacteroides_ovatus	Bacteroides_sp_1_1_6	Bacteroides_uniformis	Bacteroides_vulgatus	Prevotella_copri
0.1	3	4	3	3	0
0.5	4	4	3	3	0
1	3	4	3	3	0
2	3	4	3	4	0
5	3	10	3	4	0
10	3	54	24	18	0
100	27	99	82	81	1

Supplementary Data Table 2. Simulation of minimum number of error (False positive + False negative) the best case classifier would make using WSS method at different window sizes. Lowest error rate for given species is colored in green.

Classifier training and validation based on HMP data set.

We identified the most prevalent 21 microbial species in the FMT samples. The WSS score was calculated for all possible sample pairs of the **HMP dataset** for these 21 species (Supplementary Table 3).

The sample pairs were grouped into two classes: related (where both samples were taken from same individual at two separate times) and unrelated (where both samples came from different individuals). The related pairs were coded as 1, and unrelated sample pairs were coded as 0 (Supplementary Table 3). All sample pairs (from the HMP data set) were randomly divided randomly into training (80%) and test sets (20%). Using the training data, we built a binary classifier using Logistic regression (using Caret's package in R; https://cran.r-project.org/web/packages/caret/index.html). We used the 5-fold cross validation approach repeated 5 times to assess model validity and report the ROC (receiver operating characteristic for model optimization), and Sensitivity and Specificity along with their standard deviation (Supplementary Table 7).

- Sensitivity refers to the ratio of predicted related sample pairs vs total related sample pairs. False negative rate = 1 - Sensitivity
- Specificity refers to the ratio of predicted unrelated sample pairs vs total unrelated sample pairs. False positive rate = 1 – Specificity
- Accuracy is ratio of all correctly predicted sample pairs versus total sample pairs.

Overall, a high sensitivity rate was obtained for most species (Supplementary Table 7A). Specificity was predicted as 1.0 for 18 out of 21 species (average for all 21 species is 0.98), indicating a very low chance of misclassifying unrelated samples as related.

The best model (based on accuracy) was selected and used to predict pairwise relatedness between sample pair in the test data. We determined the accuracy, sensitivity and specificity for the predictions on the test data along with the WSS classifier cutoff (Figure 1B; Supplementary Table 7B, Supplementary Table 7C). We were able to achieve 100% classification accuracy for 17 of 21 species. The remaining four species, *B. vulgatus, B. ovatus, B. uniformis* and *E. rectale,* provided classification accuracies ranging from 0.993 to 0.999 with sensitivities ranging from 0.800 to 1.0 and specificities from 0.997-1.0 (Supplementary Table 7B – orange colored

squares). Overall, we were able to achieve a specificity of 1 (highest) for all species except *B. vulgatus*.

Identification of donor microbes in the FMT recipients.

The WSS score was calculated for all possible sample pairs for the 7 FMT cases (Supplementary Table 6). WSS was calculated for all possible sample pairs where both samples had a minimum coverage >= 20% and average read depth >= 5 for each reference species (cut-off). For our study, out of all of the samples which passed the cut-off (dots which are in the red box), 97% have coverage >40 and 95% have coverage >60 (Supplementary Data Figure 3).

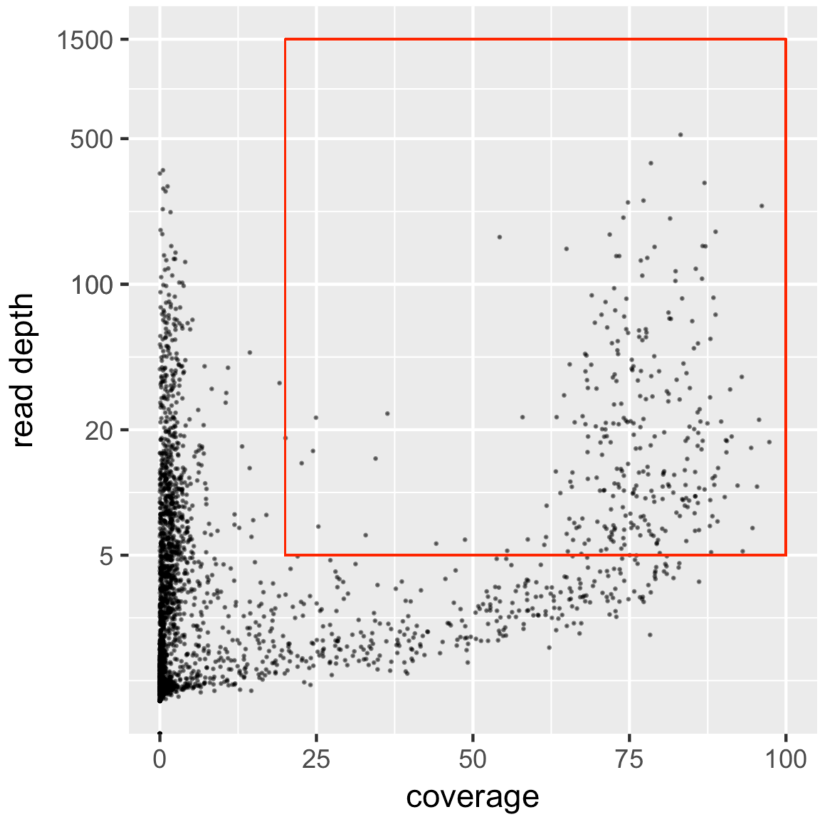

Supplementary Data Figure 3: Read depth versus genome coverage for FMT.

The sample pairs were grouped in two categories. First is the FMT-related where there is a possibility of shared microbiota (e.g. in FMTA-DA and FMTA-T1A). The second category is FMT-unrelated where two samples should not share any common microbiota (e.g. FMTB-DB and FMTA-T1A). Using the classifier based on the HMP (21 microbe species found in the FMT samples) as a training dataset, we found the FMT-related samples separated into related (represent same SNP pattern, implying presence of same species) and unrelated (the sample pair does not share same SNP pattern, implying that sample pair might have different microbes. The classifier correctly predicted that all FMT-unrelated samples (second category) were unrelated (Supplementary Table 7; Figure 2A).

Out of 77 FMT-related pairs with sufficient sequence coverage, 67 are predicted to be related based on significant WSS scores (red points in Figure 2A and red squares in Figure 2B), and 10 are predicted to be unrelated (orange points in Figure 2A and orange squares in Figure 2B). Blue points in Figure 2A correspond to FMT unrelated samples. One possible explanation for the 10 predicted to be unrelated FMT-related pairs (orange dots) is the presence of residual microbes in the recipient (i.e. sequestered such as that discussed for FMTA *F. prausnitzii*) that expand after FMT. We also cannot exclude evolution of the donor microbes in the FMT recipient that result in emergence of new strains.

Beta diversity plots for metagenomics samples.

Using the microbe abundance information (species level) from MetaPhlan2, we calculated the beta diversity (Bray-Curtis) for donor and recipient post-transplant samples and generated PCoA plot using QIIME. The pattern for each specific transplant is shown Supplementary Data Figure 4. For the transplant B, E and F the RpT samples are very close to donor signifying higher proportion of microbiome similarity as also seen in the higher number of transplanted microbes (Fig 2B). In contrast, for transplant A and G the RpT samples were more

distant to corresponding donor meaning relatively few microbes have transplanted from the donor.

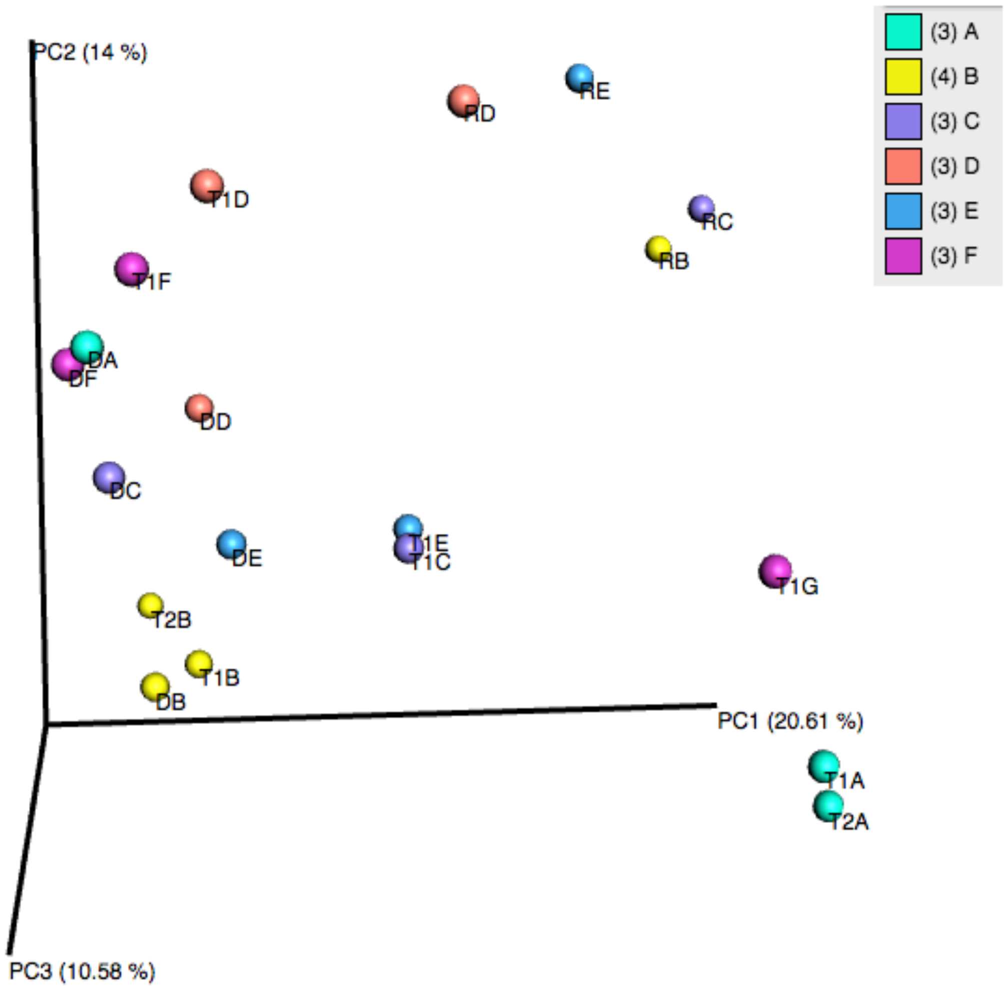

Supplementary Date Figure 4 : PCoA plot of donor and RpT samples.

References

1 Kumar, R. *et al.* Colonization potential to reconstitute a microbe community in patients detected early after fecal microbe transplant for recurrent *C. difficile*. *BMC Microbiology* **16** (2016).

2 Kumar, R. *et al.* Getting started with microbiome analysis: sample acquisition to Bioinformatics. *Curr. Protoc. Hum. Genet.* **82** (2014).

3 Segata, N. *et al.* Metagenomic microbial community profiling using unique clade-specific marker genes. *Nat Methods* **9**, 811 - 814, doi:10.1038/nmeth.2066 (2012).

4 Franzosa, E. A. *et al.* Identifying personal microbiomes using metagenomic codes. *PNAS*, E2930-E2938 (2015).
